# Challenges in the Diagnosis and Management of Immune Complex-Mediated Membranoproliferative Glomerulonephritis and Complement 3 Glomerulopathy

**DOI:** 10.1016/j.ekir.2024.09.017

**Published:** 2024-09-21

**Authors:** Andrew S. Bomback, Vivek Charu, Fadi Fakhouri

**Affiliations:** 1 Division of Nephrology, Department of Medicine, Columbia University Irving Medical Center, New York, New York, USA; 2 Department of Pathology, Stanford University, Stanford, California, USA; 3 Service of Nephrology and Hypertension, Department of Medicine, Lausanne University Hospital and University of Lausanne, Lausanne, Switzerland

**Keywords:** alternative complement pathway, complement 3 glomerulonephritis, complement 3 glomerulopathy, dense deposit disease, immune complex-mediated membranoproliferative glomerulonephritis, idiopathic immune complex-mediated membranoproliferative glomerulonephritis

## Abstract

Immune complex-mediated membranoproliferative glomerulonephritis (IC-MPGN) and complement 3 glomerulopathy (C3G) are rare, complement-mediated kidney diseases, previously classified under the group of kidney disorders termed membranoproliferative glomerulonephritis (MPGN) type 1, type 2, and type 3. Despite new advances in our understanding of IC-MPGN and C3G, several unmet needs persist in the diagnosis and management of patients with these nephropathies, due in part to their rarity and their overlapping clinical presentations, histologic features, and underlying pathophysiologies. This review summarizes our current understanding of the role of complement in IC-MPGN and C3G, and underlines the key histopathologic differences between the diseases. Using seven illustrative patient cases, we discuss consensus guideline treatment recommendations and the uncertainties, challenges, and considerations regarding the diagnosis and management of patients with IC-MPGN and C3G in clinical practice. The presented cases emphasize the need for a multidisciplinary approach encompassing primary care providers (PCPs), nephrologists, nephropathologists, and laboratory scientists. Key knowledge gaps are evaluated, including differential diagnoses, underlying pathologic mechanisms, and the lack of effective treatments targeting drivers of disease. As the therapeutic landscape evolves, an improved understanding of IC-MPGN and C3G is crucial to identifying optimal targeted-treatment strategies and facilitating a personalized approach to the management of these complex glomerular diseases.

See Commentary on Page 7

### Clinical and Histopathologic Features of IC-MPGN and C3G

IC-MPGN, also known as Ig-mediated MPGN, and C3G are rare kidney diseases.^1^^,^^2^ IC-MPGN is classified as secondary when arising from an underlying disease process.^3^ When no underlying cause can be identified, the lesion is considered “idiopathic” or “primary.”^1^^,^^3^ Although the incidence of idiopathic IC-MPGN is unknown, the estimated global annual incidence of C3G is between 1 and 3 cases per million people.^2^^,^^4^^,^^5^ The risk of progression to kidney failure is high, with up to 30% to 35% of patients with C3G and idiopathic IC-MPGN developing kidney failure within 10 years of diagnosis.^6^, ^7^, ^8^, ^9^ Both IC-MPGN and C3G fall under the group of kidney disorders termed MPGN, a pattern of glomerular injury characterized on light microscopy (LM) by an increase in mesangial cellularity and matrix, mesangial enlargement, and a thickening of the capillary walls, giving the glomerular basement membrane a “double contour” appearance.^1^^,^^3^^,^^10^

MPGN usually arises secondary to the glomerular deposition of immune complexes and/or complement factors.^1^ Historically, MPGN lesions were classified based on the location of glomerular deposits (by electron microscopy [EM]), as type 1 (subendothelial), type 2 (intramembranous), or type 3 (subendothelial and subepithelial).^10^ This classification was not optimal because it was not based on disease pathogenesis and multiple pathogenic processes fell under the type 1 and type 3 subtype designations.^1^^,^^3^^,^^10^ In 2010, a new classification based on the pathologic composition of glomerular deposits by immunofluorescence (IF) of kidney biopsies was proposed, reclassifying the MPGN subtypes into IC-MPGN and C3G (Figure 1).^16^ C3G is further divided into dense deposit disease (DDD) and C3 glomerulonephritis (C3GN).^3^ Under the new designation, the previous subtypes termed MPGN type 1 and 3 may be classified as either C3GN or IC-MPGN, and DDD replaces MPGN type 2.^1^^,^^10^ The term MPGN remains suboptimal for classification because a large proportion (up to 66%) of IC-MPGN and C3G cases do not display an MPGN pattern by LM.^12^^,^^17^, ^18^, ^19^, ^20^, ^21^, ^22^, ^23^

**Figure 1:**
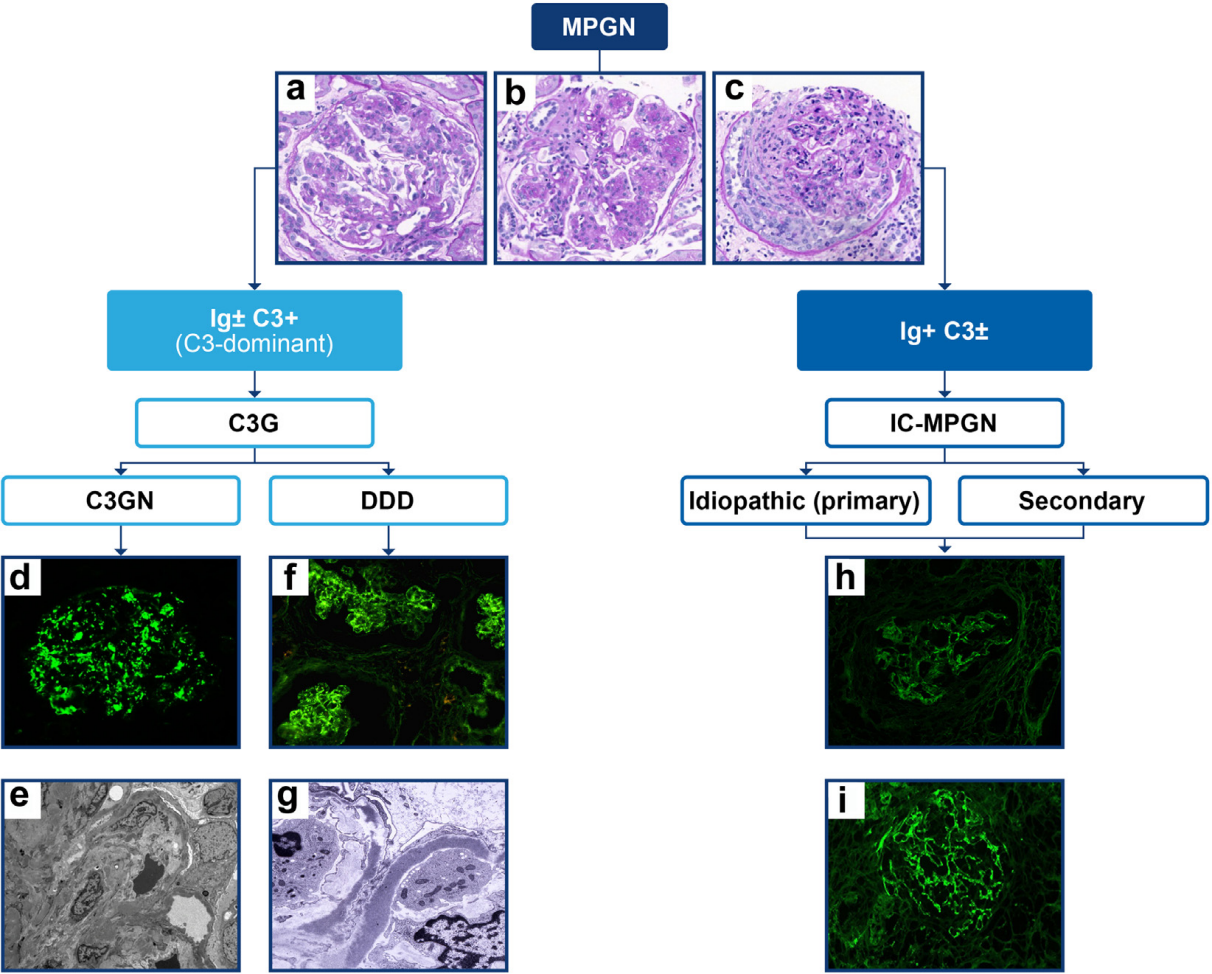
Pathobiology-based classification of membranoproliferative lesions.^3^^,^^11^ The current classification of MPGN relies on IF examination of glomerular deposits that are defined as either C3-dominant (C3G) or Ig-dominant (IC-MPGN).^3^^,^^11^ Other unrelated disorders may present with an MPGN pattern of injury in the absence of C3 and Ig deposits, such as chronic thrombotic microangiopathy, aHUS, and chronic transplant glomerulopathy (not shown).^1^^,^^3^ (a) Mesangioproliferative injury; (b) classical membranoproliferative injury with lobular proliferation and double contours in basement membranes; (c) crescentic injury; (d) C3GN with intense granular deposits of C3 in mesangial regions and segmental deposits along the capillary walls; (e) C3GN with electron-dense deposits in subendothelial, intramembranous, and mesangial regions viewed by EM; (f) DDD with mesangial and capillary staining for C3; (g) DDD with ribbon-like deposits along the basement membrane lamina densa; (h) IC-MPGN with IgG staining; and (i) IC-MPGN with C3 staining. aHUS, atypical hemolytic uremic syndrome; C, complement; C3G, complement 3 glomerulopathy; C3GN, complement 3 glomerulonephritis; DDD, dense deposit disease; EM, electron microscopy; IC-MPGN, immune complex-mediated membranoproliferative glomerulonephritis; IF, immunofluorescence; MPGN, membranoproliferative glomerulonephritis. Images reprinted from Kovala *et al.*,^12^ Li *et al.*,^13^ Ponticelli *et al.*,^14^ and Hanna *et al.*^15^ without modification under the terms and conditions of the Creative Commons Attribution (CC BY) license (https://creativecommons.org/licenses/by/4.0/).

Under the new classification, C3G is defined by C3 IF staining of ≥2 orders of intensity stronger than any other immune reactant, with little or no Ig deposits, whereas IC-MPGN is characterized by glomerular deposits of immune complexes containing both Ig and complement proteins.^3^^,^^24^ Differential diagnosis of C3GN and DDD is based on the ultrastructural appearance of electron-dense deposits by EM.^1^^,^^25^ DDD is characterized by highly electron-dense osmiophilic deposits with a “ribbon-like” appearance in the glomerular basement membrane; in comparison, C3GN exhibits less-dense deposits, which are usually amorphous or cloudy in appearance within the mesangium and can appear as ill-defined subendothelial inclusions.^1^^,^^25^ Recent evidence from a study of 12 patients with C3GN and DDD suggests that dense deposits in DDD are enriched in apolipoprotein E compared with C3GN, and that apolipoprotein E staining may augment the differentiation of C3GN and DDD.^26^

### The Role of the Complement System in IC-MPGN and C3G Pathophysiology

IC-MPGN and C3G share overlapping pathologic features, with dysregulation of the complement system playing a key role in the pathogenesis of both diseases. Overactivation of the alternative complement pathway (AP) is the primary driver of C3G pathophysiology, which leads to accumulation of C3 in the glomerulus, resulting in kidney inflammation and damage.^2^^,^^18^^,^^25^ In many patients, uncontrolled activation of the AP in C3G is driven by genetic abnormalities in key AP genes and/or acquired autoantibodies against complement proteins such as the C3 and C5 nephritic factors that stabilize the AP C3 and C5 convertases, respectively, thereby prolonging their half-life (Table 1).^2^^,^^27^

**Table 1:** Frequency of genetic and acquired abnormalities in complement proteins in patients with IC-MPGN and C3G from published cohort studies

Complement protein(s)	Reference	C3G, *n/N* (%)	C3GN, *n/N* (%)	DDD, *n/N* (%)	IC-MPGN, *n/N* (%)
Complement factor gene variant or mutation					
*C3*	Bomback *et al.*^19^		2/42 (4.8)	0/9 (0)	
	Ravindran *et al.*^21^	6/62 (9.7)			
	Ravindran *et al.*^20^		6/61 (9.8)		
	Garam *et al.*^39^		1/37 (2.7)	1/11 (9.1)	2/44 (4.5)^a^
	Iatropoulos *et al.*^7^		6/50 (12.0)	0/21 (0)	6/64 (9.4)^a^
	Iatropoulos *et al.*^8^		9/68 (13.2)	0/25 (0)	5/80 (6.3)^a^
*CFB*	Bomback *et al.*^19^		0/42 (0)	0/9 (0)	
	Ravindran *et al.*^21^	1/62 (1.6)			
	Garam *et al.*^39^		1/37 (2.7)	0/11 (0)	0/44 (0)^a^
	Iatropoulos *et al.*^7^		0/50 (0)	0/21 (0)	3/64 (4.7)^a^
	Iatropoulos *et al.*^8^		0/68 (0)	0/25 (0)	3/80 (3.8)^a^
*CFH*	Servais *et al.*^6^		9/56 (16.1)	5/29 (17.2)	
	Bomback *et al.*^19^		8/42 (19.0)	3/9 (33.3)	
	Ravindran *et al.*^21^	10/62 (16.1)			
	Ravindran *et al.*^20^		8/61 (13.1)	2/9 (22.2)	
	Garam *et al.*^39^		1/37 (2.7)	1/11 (9.1)	1/44 (2.3)^a^
	Iatropoulos *et al.*^7^		3/50 (6.0)	1/21 (4.8)	1/64 (1.6)^a^
	Iatropoulos *et al.*^8^		5/68 (7.4)	2/25 (8.0)	3/80 (3.8)^a^
*CFHR5*	Bomback *et al.*^19^		1/42 (2.4)	0/9 (0)	
	Ravindran *et al.*^21^	4/62 (6.5)			
	Ravindran *et al.*^20^		4/61 (6.6)		
*CFI*	Servais *et al.*^6^		4/56 (7.1)	2/29 (6.9)	
	Bomback *et al.*^19^		0/42 (0)	0/9 (0)	
	Ravindran *et al.*^21^	2/62 (3.2)			
	Ravindran *et al.*^20^		2/61 (3.3)		
	Garam *et al.*^39^		1/37 (2.7)	1/11 (9.1)	0/44 (0)^a^
	Iatropoulos *et al.*^7^		1/50 (2.0)	1/21 (4.8)	0/64 (0)^a^
	Iatropoulos *et al.*^8^		3/68 (4.4)	1/25 (4.0)	0/80 (0)^a^
*MCP*	Servais *et al.*^6^		2/56 (3.6)	0/29 (0)	
	Bomback *et al.*^19^		1/42 (2.4)	0/9 (0)	
	Ravindran *et al.*^21^	0/21 (0)^b^			
	Garam *et al.*^39^		4/37 (10.8)	0/11 (0)	4/44 (9.1)^a^
	Iatropoulos *et al.*^7^		0/50 (0)	0/21 (0)	1/64 (1.6)^a^
	Iatropoulos *et al.*^8^		0/68 (0)	0/25 (0)	1/80 (1.3)^a^
*THBD*	Garam *et al.*^39^		2/37 (5.4)	0/11 (0)	2/44 (4.5)^a^
	Iatropoulos *et al.*^7^		0/50 (0)	1/21 (4.8)	0/64 (0)^a^
	Iatropoulos *et al.*^8^		1/68 (1.5)	1/25 (4.0)	0/80 (0)^a^
Complement-associated autoantibody					
C3NeF	Servais *et al.*^6^		24/53 (45.3)	19/22 (86.4)	
	Bomback *et al.*^19^		13/42 (31.0)	1/9 (11.1)	
	Ravindran *et al.*^21^	25/61 (41.0)			
	Ravindran *et al.*^20^	30/69 (43.5)	27/59 (45.8)	3/10 (30.0)	
	Garam *et al.*^39^			5/11 (45.5)	11/44 (25.0)^a^
	Garam *et al.*^38^		7/40 (17.5)	5/12 (41.6)	15/67 (22.4)^a^
	Iatropoulos *et al.*^7^	NA/NA (54)	NA/NA (44)	NA/NA (78)	NA/NA (44)^a^
	Iatropoulos *et al.*^8^		24/62 (38.7)	18/23 (78.3)	31/77 (40.3)^a^
	Kovala *et al.*^12^	2/11 (18.2)			0/22 (0)^c^
C4NeF	Garam *et al.*^38^		7/40 (17.5)	1/12 (8.3)	9/67 (13.4)^a^
C5NeF	Ravindran *et al.*^20^		1/59 (1.7)		
Factor B	Bomback *et al.*^19^		0/42 (0)	0/9 (0)	
	Ravindran *et al.*^21^	5/60 (8.3)			
	Ravindran *et al.*^20^		5/59 (8.5)		
	Garam *et al.*^38^		3/37 (8.1)	2/12 (16.7)	2/67 (3.0)^a^
	Kovala *et al.*^12^	2/13 (15.4)			1/17 (5.9)^c^
Factor H	Bomback *et al.*^19^		3/42 (7.1)	1/9 (11.1)	
	Ravindran *et al.*^21^	3/60 (5.0)			
	Ravindran *et al.*^20^		2/59 (3.4)	1/8 (12.5)	
	Kovala *et al.*^12^	1/13 (7.7)			1/18 (5.6)^c^

C, complement; C3G, complement 3 glomerulopathy; C3GN, complement 3 glomerulonephritis; CFB, complement factor B; CFH, complement factor H; CFHR5, complement factor H related 5; CFI, complement factor I; DDD, dense deposit disease; IC-MPGN, immune complex-mediated membranoproliferative glomerulonephritis; MCP, membrane cofactor protein; NA, not available; NeF, nephritic factor; THBD, thrombomodulin.

a Patients with idiopathic IC-MPGN only (patients with secondary IC-MPGN were excluded from the study).

b Patients with C3G and monoclonal Ig only.

c Includes patients with primary and secondary IC-MPGN.

IC-MPGN can occur secondary to various underlying diseases such as infection or autoimmune conditions.^1^^,^^3^ Some cases of secondary IC-MPGN and C3G are also associated with monoclonal gammopathies,^3^^,^^21^^,^^28^, ^29^, ^30^, ^31^, ^32^ which are encompassed by the spectrum of disorders called monoclonal gammopathies of renal significance.^33^^,^^34^ The role of the complement system in the pathogenesis of IC-MPGN is less well-understood, and mechanisms leading to the deposition of immune complexes remain unknown.^35^ Immune complexes in both idiopathic and secondary IC-MPGN activate the classical complement pathway,^36^^,^^37^ whereas several studies have reported similar levels of genetic and/or acquired AP abnormalities in patients with idiopathic IC-MPGN and C3G (Table 1).^7^^,^^8^^,^^38^^,^^39^ In addition, the prevalence of low serum C3 with normal serum C4, indicative of AP activation, is comparable in patients with idiopathic IC-MPGN and C3G.^7^^,^^8^^,^^39^ This highlights a role for overactivation of the AP in idiopathic IC-MPGN.

### Uncertainties and Unmet Needs in IC-MPGN and C3G

Despite recent experimental and clinical work, several uncertainties remain concerning the diagnosis and management of IC-MPGN and C3G. Patients with these disorders may exhibit similar clinical characteristics at disease onset, such as proteinuria, hematuria, decreased kidney function, nephrotic syndrome, and hypertension^7^^,^^8^^,^^12^^,^^38^, ^39^, ^40^; definitive diagnosis therefore requires a kidney biopsy.^3^^,^^24^^,^^35^ However, distinguishing IC-MPGN and C3G by kidney biopsy remains a challenge, due to overlap in the composition of glomerular deposits and the subjective grading of IF staining intensity.^25^^,^^35^^,^^41^^,^^42^ In addition, consecutive biopsies in the same patient may reveal a change in IF pattern from IC-MPGN to C3G, or vice versa, depending on the time course of the disease.^43^^,^^44^ Beyond the complexities in diagnosis, an additional key barrier in the management of idiopathic IC-MPGN and C3G is the current lack of treatments that target the pathologic mechanisms of these diseases.^45^

To highlight the challenges and uncertainties in the diagnosis and management of patients with IC-MPGN and C3G, we present a series of illustrative cases that reflect patients encountered in clinical practice across the continuum of these nephropathies (Figure 2). In the context of these cases, we review best practices in the diagnostic workflow, discuss current evidence in patient management, and examine unmet needs.

**Figure 2:**
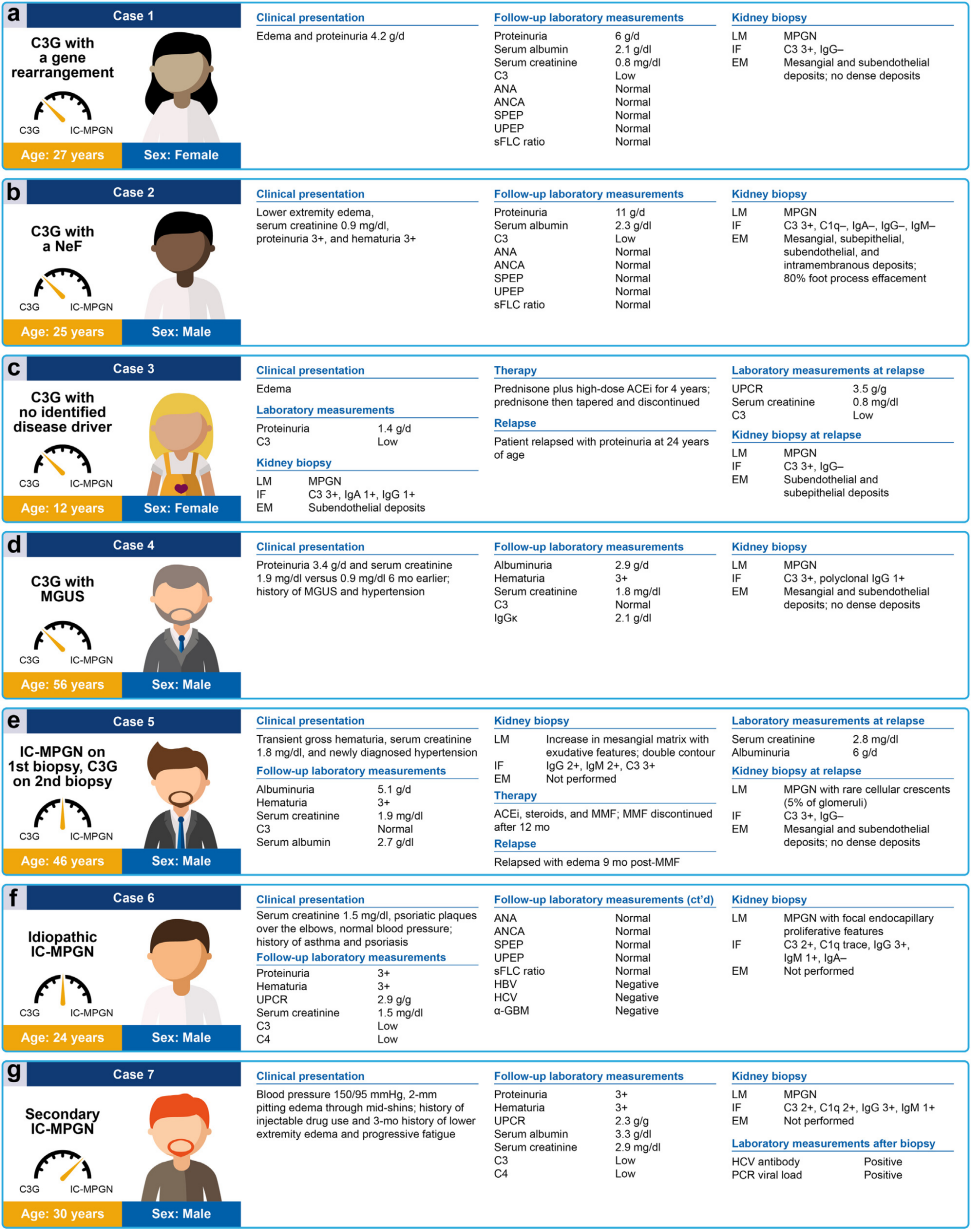
Illustrative clinical cases involving patients with IC-MPGN or C3G. α-GBM, antiglomerular basement membrane antibody; ACEi, angiotensin-converting enzyme inhibitor; ANA, antinuclear antibody; ANCA, antineutrophil cytoplasmic antibody; C, complement; C3G, complement 3 glomerulopathy; EM, electron microscopy; HBV, hepatitis B virus; HCV, hepatitis C virus; IC-MPGN, immune complex-mediated membranoproliferative glomerulonephritis; IF, immunofluorescence; LM, light microscopy; MGUS, monoclonal gammopathy of unknown significance; MMF, mycophenolate mofetil; MPGN, membranoproliferative glomerulonephritis; NeF, nephritic factor; PCR, polymerase chain reaction; sFLC, serum free light chains; SPEP, serum protein electrophoresis; UPCR, urine protein-to-creatinine ratio; UPEP, urine protein electrophoresis.

### Case 1: C3G With a Gene Rearrangement

A 27-year-old female patient was referred to the nephrologist for edema and nephrotic range proteinuria (4.2 g/d) initially detected on urinalysis conducted by the patient’s PCP (Figure 2a). Repeat laboratory tests revealed proteinuria 6 g/d on 24-hour collection, low serum albumin, normal serum creatinine, and low C3, with otherwise normal serologies (antinuclear antibody, antineutrophil cytoplasmic antibody, serum protein electrophoresis, urine protein electrophoresis, and serum free light chains ratio). A biopsy showed MPGN by LM, C3 deposits only by IF, and mesangial and subendothelial deposits without dense deposits by EM. The patient was given a pathologic diagnosis of C3GN. Testing for genetic variants and autoantibodies associated with AP hyperactivity revealed no autoantibodies but identified the presence of a mutation in CFHR1, resulting in a duplication of its dimerization domain.

#### Case Highlights and Practical Considerations

Up to 40% of patients with C3GN and up to 33% of patients with DDD have detectable genetic abnormalities in AP genes such as *C3*, *CFB*, *CFH*, *CFHR*, *CFI*, *MCP* (*CD46*), and *THBD*, including variants of unknown significance (Table 1).^6^, ^7^, ^8^^,^^19^^,^^20^^,^^38^^,^^39^ Rare pathogenic, or likely pathogenic, variants in genes of the AP are present in a much smaller proportion of patients with C3G (∼17%–35%)^7^^,^^12^^,^^20^^,^^38^^,^^39^; the presence of these variants in patients with atypical hemolytic uremic syndrome is higher, at approximately 51%.^46^ Nevertheless, the presence of AP factor gene variants in C3G illustrates the role of AP overactivation in disease pathogenesis, and highlights the importance of complement genetic testing in all patients after a pathologic diagnosis of C3G.^35^ However, the impact of AP factor gene variants on patient outcomes in C3G is unclear,^41^ and there is limited choice in currently available therapies.^3^^,^^45^ The 2021 Kidney Disease: Improving Global Outcomes guidelines recommend mycophenolate mofetil (MMF) plus glucocorticoids, in addition to supportive care, as first-line therapy in patients with moderate-to-severe disease and the absence of monoclonal gammopathy, with consideration of an anti-C5 antibody in patients who fail treatment, followed by enrollment in a clinical trial.^3^ No randomized, prospective clinical trials have confirmed the benefit of these nonspecific treatments. Further, no agents targeting components of the AP are currently approved for the treatment of C3G, but several complement inhibitors are in clinical development.^47^, ^48^, ^49^, ^50^, ^51^, ^52^, ^53^, ^54^ In the future, the presence or absence of constitutive overactivation of the AP may help determine the optimal duration of therapeutic complement inhibition, as illustrated in atypical hemolytic uremic syndrome, for which anti-C5 antibodies have been approved for several years.^55^

### Case 2: C3G With a NeF

A 25-year-old male patient noticed lower extremity edema and saw their PCP, with laboratory tests showing normal serum creatinine but proteinuria 3+ and hematuria 3+ on urinalysis (Figure 2b). The patient was referred to a nephrologist, who ordered repeat laboratory tests; these tests revealed proteinuria 11 g/d on 24-hour collection, low serum albumin, and low C3 level with otherwise normal serologies (antinuclear antibody, antineutrophil cytoplasmic antibody, serum protein electrophoresis, urine protein electrophoresis, and serum free light chains ratio). A biopsy showed MPGN by LM; with IF staining showing C3 3+ and negative C1q, IgG, IgM, and IgA. EM revealed mesangial, subepithelial, subendothelial, and intramembranous deposits, and 80% foot process effacement. The patient was given a pathologic diagnosis of C3GN and was enrolled in a research study on the natural history of C3GN, which included access to testing for genetic variants and autoantibodies associated with AP hyperactivity. This workup revealed no pathogenic genetic variants but was positive for a C3NeF.

#### Case Highlights and Practical Considerations

The detection of autoantibodies in patients with C3G (up to 46% of C3GN and up to 86% of DDD) is more likely than the detection of pathogenic gene variants.^6^, ^7^, ^8^^,^^19^^,^^20^^,^^22^^,^^38^^,^^39^ C3NeF is the most common autoantibody detected, but autoantibodies that stabilize the C3 convertase of the lectin pathway and classical complement pathway (C4 nephritic factors), or those that recognize other complement proteins including C1q, C3, factor H, and factor B, have also been documented (Table 1).^12^^,^^19^, ^20^, ^21^, ^22^^,^^27^^,^^38^^,^^56^^,^^57^ Fluctuation of C3 nephritic factors has been reported but has not been correlated with disease activity in patients with C3G.^6^^,^^58^

The association of C3NeF positivity with improved patient outcomes and greater response to immunosuppressive therapy, including MMF-based treatment, has been examined in several studies^19^^,^^22^^,^^59^; however, evidence to date has been derived from small, retrospective, nonrandomized studies that did not account for all potential confounders, particularly inflammatory changes. In a study that included 60 patients with C3GN,^59^ a higher proportion of C3NeF-positive patients (8/11; 80%) showed remission after immunosuppressive therapy compared with C3NeF-negative patients (3/12; 37%). In addition, a lower proportion progressed to kidney failure (2/11 [20%] and 5/12 [63%], respectively). However, these differences did not reach statistical significance, and histologic changes were not assessed. In a United States cohort of 42 patients with C3G who were treated with MMF,^19^ the prevalence of C3NeF positivity was numerically higher in MMF responders (4/9; 44%) than in MMF nonresponders (4/14; 29%); however, no statistical analyses were reported and the study investigators concluded there were no clear predictors of response to treatment. Finally, in a study of 97 patients with C3G treated with MMF plus corticosteroids, other immunosuppressants, an anti-C5 antibody, or other conservative management therapies,^22^ patients who achieved complete remission across all treatment subgroups had a significantly higher percentage of C3NeF (8/18; 44%) compared with patients with partial remission (3/28; 11%). Among the 29 patients who were positive for complement autoantibodies, treatment with MMF plus corticosteroids was associated with a significantly lower probability of kidney failure within 10 years compared with the other therapies. Nonetheless, based on current evidence the optimal treatment strategy for patients with autoantibody-positive C3G remains unclear.

### Case 3: C3G With No Identified Disease Driver

A 12-year-old female patient presented to their pediatrician with edema and was found to have microscopic hematuria, proteinuria 1.4 g/d on 24-hour collection, and low C3 level after workup by pediatric nephrology (Figure 2c). The patient underwent a kidney biopsy, which showed MPGN features by LM; the presence of C3 3+, IgG 1+, and IgA 1+ by IF; and subendothelial deposits by EM. The biopsy was read as MPGN type 1 and the patient was treated for 4 years with prednisone and a high-dose angiotensin-converting enzyme inhibitor. The prednisone was tapered and discontinued after 4 years, and the patient had sustained remission of proteinuria until they were aged 24 years, when they relapsed and were seen by a new nephrologist. Laboratory tests showed preserved kidney function but a low C3 level and an elevated urine protein-to-creatinine ratio of 3.5 g/g. A repeat biopsy was performed and was consistent with C3G, showing an MPGN pattern by LM, C3 deposits only by IF, and subendothelial and subepithelial deposits by EM. At this time, pathology reviewed the patient’s prior biopsy, which was reclassified as C3G. The patient underwent a commercially available genetics panel geared toward atypical hemolytic uremic syndrome, with no pathogenic lesions identified, and had C3NeF checked as part of routine laboratory work, which was also negative.

#### Case Highlights and Practical Considerations

This patient is typical of a number of cases of C3G where the patient was diagnosed as MPGN type 1, 2, or 3 prior to the reclassification of MPGN into IC-MPGN and C3G, who in retrospect would have been diagnosed with C3G under the new criteria. In this case, a repeat biopsy brought to light the clearer diagnosis.

Many cases of C3G (∼35%–87% in published cohorts) do not have a detectable autoantibody and/or an identified genetic variant in the AP,^7^^,^^19^^,^^39^ although it is worth noting that patients with C3G may carry genetic variants of unknown significance.^20^^,^^60^ Genetic testing can miss unique or pathogenic variants,^61^ and the applicability of sequencing in the identification of rare complement gene variants remains unclear. Levine *et al.*^62^ reported no enrichment of rare variants in complement genes or the whole exome among 146 cases of primary MPGN, including 53 cases of idiopathic IC-MPGN and 61 cases of C3G, compared with controls. Nevertheless, the lack of an identified disease driver does not preclude patients with C3G from participating in clinical trials of targeted complement inhibitors, and current Kidney Disease: Improving Global Outcomes guidelines recommend that patients with moderate-to-severe C3G who fail to respond to MMF plus glucocorticoids and an anti-C5 antibody be considered for a clinical trial, where available.^3^

Increasingly, a role for autoimmunity has been implicated in the development of both C3G and IC-MPGN. In the aforementioned study by Levine *et al.*,^62^ a significant increase in the prevalence of human leukocyte antigen serotypes DQ2, B8, and DR17 was identified in patients with idiopathic IC-MPGN and C3G compared with controls, suggesting that autoimmune processes may play a role in disease development. In a subsequent study, human leukocyte antigen-DR17 was associated with kidney failure in C3GN and IC-MPGN, but not in DDD.^63^

### Case 4: C3G With Monoclonal Gammopathy of Unknown Significance

A 56-year-old male patient with a history of hypertension and monoclonal gammopathy of unknown significance underwent a regular check-up with their PCP (Figure 2d). Laboratory tests showed increased serum creatinine (1.9 mg/dl vs. 0.9 mg/dl 6 months earlier), with proteinuria 3.4 g/d. The patient was referred to a nephrologist. Additional workup confirmed elevated serum creatinine, albuminuria (2.9 g/d), and hematuria 3+ with persistent monoclonal gammopathy (IgGκ); the patient’s C3 serum level was normal. Testing for genetic variants and autoantibodies associated with AP hyperactivity was negative. A biopsy showed MPGN by LM, with predominant C3 (3+) deposits by IF (polyclonal IgG 1+), and EM showed mesangial and subendothelial deposits without dense deposits.

#### Case Highlights and Practical Considerations

Monoclonal gammopathy is frequently detected in patients with C3G who are aged ≥50 years.^21^^,^^30^, ^31^, ^32^ However, the mechanism linking monoclonal gammopathy to complement dysregulation remains poorly understood.^13^^,^^30^^,^^31^ Pathogenic variants in AP genes are rarely detected in patients with monoclonal gammopathy–associated C3G^21^^,^^64^; rather, monoclonal Ig (MIg) may lead to overactivation of the AP by directly activating C3 convertase or by acting as autoantibodies against other regulatory complement proteins.^21^^,^^28^^,^^64^ In a French cohort including 50 patients with monoclonal gammopathy-associated C3G, treatment with anti B-cell chemotherapy was associated with significantly higher kidney responses and kidney survival rates in patients who achieved a hematologic response compared with those receiving conservative or immunosuppressive therapy, indirectly suggesting that MIg plays a pathogenic role in C3 deposition.^32^ In the same study, MIg had prognostic relevance, with the presence of serum, urine, and/or glomerular MIg associated with shorter kidney survival compared with 26 patients with C3G who were aged >50 years without MIg.^32^ Nevertheless, the prognostic significance of MIg remains unclear; a United States study including 36 and 59 patients with C3G with and without MIg, respectively, showed no statistically significant difference in kidney survival between the patient groups.^21^ In addition, kidney survival was comparable between patients who did and did not receive monoclonal protein-targeted therapy.^21^ Traditional therapies for C3G, such as MMF and steroids, have shown favorable outcomes in MIg-associated disease,^65^ and further study is needed to determine if this form of C3G responds to novel, targeted complement inhibitors.

### Case 5: IC-MPGN on First Biopsy but C3G on Second Biopsy

A 46-year-old male patient was referred to the nephrology department by their PCP for newly diagnosed hypertension, transient gross hematuria, and elevated serum creatinine (Figure 2e). The workup performed by the nephrologist confirmed elevated serum creatinine, low serum albumin, albuminuria 5.1 g/d, and hematuria 3+ by dipstick. A kidney biopsy showed an increase in the mesangial matrix with exudative features (inflammatory cells in glomerular capillaries) and double contour by LM; and the presence of IgG 2+, IgM 2+, and C3 3+ deposits by IF. An EM analysis was not performed. The patient’s serum C3 level was normal. The patient was evaluated for secondary etiologies, including infection, autoimmune disease, and malignancy (all of which were negative), and was given a diagnosis of idiopathic IC-MPGN. The patient received an angiotensin-converting enzyme inhibitor, steroids, and MMF. Albuminuria decreased to 1.2 g/d, serum albumin normalized, and serum creatinine stabilized at 1.8 mg/dl. MMF was discontinued after 12 months of treatment. Nine months later, the patient presented with edema, heavy albuminuria 6 g/d, and worsening renal function (serum creatinine 2.8 mg/dl). A second biopsy showed MPGN features with rare cellular crescents (5% of glomeruli) by LM, exclusive C3 3+ deposits by IF (IgG−), and EM showed mesangial and subendothelial deposits, without dense deposits. The patient had no clinical or biological features of infection.

#### Case Highlights and Practical Considerations

Whether idiopathic IC-MPGN and C3G are distinct entities or two aspects of the same disease remains a debated question, because C3G and idiopathic IC-MPGN exhibit similar clinical presentations and patient outcomes, including similar risk of progression to kidney failure and prevalence of nephrotic syndrome.^7^^,^^8^^,^^38^^,^^39^ Kidney pathologic features on LM and IF may vary from one biopsy to another in the same patient throughout the disease course; in a cohort study of patients with primary MPGN who had archived kidney biopsies, 10 of 23 patients (43%) with repeat biopsies had different IF staining patterns on their follow-up biopsies, and 4 of 23 (17%) exhibited a shift from C3G to idiopathic IC-MPGN or vice versa.^43^ This suggests that some cases of idiopathic IC-MPGN may fall in the same disease continuum as C3G. Switching has been reported in the pediatric population, where evidence suggests that a subgroup of children can initially present with a biopsy consistent with IC-MPGN but show dominant C3 in a subsequent biopsy.^44^ This may be caused by an infectious trigger at onset that activates the lectin pathway, classical complement pathway, and AP, with the classical complement pathway becoming less active as the infection resolves and/or due to immunosuppression.^44^

Known drivers of AP overactivation (a key feature of many cases of C3G)^2^^,^^27^ have been found in some patients with idiopathic IC-MPGN, providing further evidence for a clinical overlap between idiopathic IC-MPGN and C3G. Indeed, several studies have reported similar levels of genetic abnormalities in key AP genes and/or acquired autoantibodies against complement proteins in patients with idiopathic IC-MPGN and C3G (Table 1).^7^^,^^8^^,^^38^^,^^39^ In addition, the prevalence of low serum C3 with normal serum C4, indicative of AP activation, is comparable in patients with idiopathic IC-MPGN and C3G.^6^, ^7^, ^8^^,^^39^

With respect to currently available therapies, there is little evidence that responses to treatments are different among patients with IC-MPGN and C3G.^35^ Current treatments primarily target inflammation, and the benefit of MMF and corticosteroids in IC-MPGN is suggested by extrapolation from retrospective studies in C3G.^35^^,^^59^^,^^66^ However, the long-term benefit of corticosteroids and immunosuppressive therapies is uncertain.^45^ Ongoing interventional clinical trials will help determine whether idiopathic IC-MPGN and C3G respond similarly to targeted complement inhibitors.^47^, ^48^, ^49^, ^50^, ^51^, ^52^, ^53^, ^54^^,^^67^^,^^68^

### Case 6: Idiopathic (Primary) IC-MPGN

A 24-year-old male patient with a past medical history notable only for asthma and psoriasis was referred to nephrology after laboratory tests showed elevated serum creatinine of 1.5 mg/dl (Figure 2f). The patient’s examination was unremarkable apart from psoriatic plaques over the elbows; blood pressure was normal. Repeat bloodwork confirmed elevated creatinine at 1.5 mg/dl, with decreased C3 and C4 levels, hematuria 3+, proteinuria 3+, and a spot urine protein-to-creatinine ratio of 2.9 g/g. Serologies, including antinuclear antibody, antineutrophil cytoplasmic antibody, antiglomerular basement membrane antibody, hepatitis B virus, hepatitis C virus, serum protein electrophoresis, urine protein electrophoresis, and serum free light chains ratio were negative or normal. A biopsy showed MPGN with focal endocapillary proliferative features, with IF staining showing IgM 1+, IgG 3+, negative IgA, C3 2+, and trace C1q.

#### Case Highlights and Practical Considerations

The presence of immune complexes containing both polyclonal Ig and complement proteins is a hallmark of IC-MPGN.^3^^,^^69^ To rule out secondary causes of disease, the diagnostic workup in patients with IC-MPGN should include evaluation of infection, autoimmunity, and underlying malignancies.^3^ In patients for whom the workup is negative, IC-MPGN is considered idiopathic or primary.^3^ It has been suggested that in some cases a diagnosis of idiopathic IC-MPGN represents secondary IC-MPGN, in which an underlying cause could not be identified.^11^ Nevertheless, the presence of genetic and/or acquired AP abnormalities in IC-MPGN without an identifiable secondary cause supports the existence of a subgroup of idiopathic cases.^7^^,^^8^^,^^38^^,^^39^

The optimal treatment pathway for patients with idiopathic IC-MPGN remains to be defined, and few clinical trials have been conducted in this population since the reclassification of MPGN.^3^ In most cases, Kidney Disease: Improving Global Outcomes guidelines recommend treatment with supportive care and carefully considered use of conventional immunosuppression, followed by enrollment in a clinical trial if appropriate.^3^ To date, few interventional studies have evaluated the potential benefits of selective complement inhibitors in idiopathic IC-MPGN, which in part reflects the rare nature of the disease and the incomplete understanding of its natural clinical course.^45^^,^^67^ Nonetheless, interventional clinical trials exploring the efficacy of complement inhibitors in idiopathic IC-MPGN are ongoing, and may facilitate the adoption of novel therapies.^50^^,^^67^^,^^68^

### Case 7: Secondary IC-MPGN

A 30-year-old male patient was evaluated at the hospital for elevated creatinine after being admitted for a 3-month history of lower extremity edema and progressive fatigue (Figure 2g). The patient had a history of injectable drug use. On physical examination, blood pressure was 150/95 mmHg; other vital signs were normal. The patient had 2-mm pitting edema through the midshins, without rash or joint findings. Laboratory tests showed serum creatinine 2.9 mg/dl, low C3 and C4 levels, serum albumin 3.3 g/dl, urinalysis with proteinuria 3+ and hematuria 3+, and a spot urine protein-to-creatinine ratio of 2.3 g/g. A kidney biopsy showed MPGN by LM; and IF showed IgG 3+, IgM 1+, C1q 2+, and C3 2+. Hepatitis C virus antibody returned positive, with follow-up positive viral load by polymerase chain reaction.

#### Case Highlights and Practical Considerations

As with all immune complex-mediated glomerulonephritides, a diagnosis of IC-MPGN should elicit a workup for known secondary causes, including systemic and drug-induced autoimmune disease (e.g., systemic lupus erythematosus, Sjögren’s syndrome, rheumatoid arthritis, and mixed connective tissue disease), infectious agents (e.g., hepatitis C virus, hepatitis B virus, bacterial, or parasitic infections), and hematologic malignancy characterized by the deposition of MIg or paraproteins due to monoclonal gammopathies (e.g., cryoglobulinemia).^1^^,^^3^^,^^70^, ^71^, ^72^ Initial treatment of secondary IC-MPGN should focus on the underlying disease process, highlighting the importance of careful testing for known drivers of secondary IC-MPGN in the diagnostic workup; in addition, routine care for patients with active glomerular disease is recommended.^3^ In the era of direct-acting antiviral medications, patients with hepatitis C virus-associated IC-MPGN and stable kidney function in the absence of nephrotic syndrome can often be treated with antiviral medications alone.^73^ Antiviral medication in combination with immunosuppression, with or without plasma exchange, may be required if the biopsy or clinical presentation is consistent with rapidly progressive glomerulonephritis or cryoglobulinemic flare.^73^

### Summary and Conclusion

In recent years, the classification of kidney disorders exhibiting an MPGN pattern of glomerular injury has evolved into IC-MPGN and C3G. The two diseases are differentiated by glomerular deposits of C3 in the presence or absence of immune complexes, respectively, determined by IF on kidney biopsy.^3^^,^^10^^,^^11^ Despite the poor outcomes and high risk of kidney failure for patients with IC-MPGN and C3G, there is a lack of high-quality evidence in the recommendations for their management, and several unmet needs persist in the diagnosis and treatment of patients with these nephropathies.^3^^,^^35^

Diagnosis of patients with IC-MPGN and C3G remains a challenge, partly due to their overlapping clinical presentations and histologic features. Therefore, definitive diagnosis relies on kidney biopsy accompanied by IF microscopy.^3^^,^^24^^,^^35^ However, histologic examination is confounded by overlap in the composition of glomerular deposits,^3^^,^^35^^,^^41^^,^^42^^,^^74^ a need for further definition of diagnostic criteria,^3^ and, in some cases, the variation of kidney pathologic features throughout the disease course.^43^^,^^44^ Therefore, histologic testing alone does not suffice, and examination for underlying etiologies and differential diagnoses, as well as a comprehensive complement workup to identify underlying genetic or acquired abnormalities in complement proteins in C3G, are crucial for a precise diagnosis and to guide treatment decisions.^3^^,^^35^ Whereas overactivation of the AP is the underlying cause of C3G,^2^^,^^18^^,^^25^ the role of the complement system in the pathogenesis of IC-MPGN is less clear, particularly with respect to the idiopathic form. Therefore, further in-depth study of the role of complement, as well as the mechanisms leading to deposition of immune complexes, is required.^35^^,^^62^

A key barrier in the management of patients with IC-MPGN and C3G is a lack of effective and targeted treatments that modify the pathophysiological drivers of disease. In part, this has been due to previous randomized controlled trials including patients based on a now discarded EM classification, rather than the current IF-based classification^3^; in addition, the rarity of patients with IC-MPGN and C3G hinders recruitment for clinical trials to test new therapies. Therefore, depending on the severity of proteinuria and kidney dysfunction, current therapeutic strategies focus primarily on supportive care and nonspecific immunosuppression, although further clinical trials are needed to assess the value of immunosuppression in idiopathic IC-MPGN and C3G.^3^ Nevertheless, the future treatment landscape for idiopathic IC-MPGN and C3G holds promise, with ongoing trials focusing on targeted therapies aimed at modulating overactivation of the complement system.^47^, ^48^, ^49^, ^50^, ^51^, ^52^, ^53^, ^54^^,^^67^^,^^68^ These targeted treatments may potentially offer more tailored and effective therapeutic options.

In summary, the diagnosis and management of IC-MPGN and C3G requires a comprehensive approach utilizing kidney biopsy, IF microscopy, complement workup, genetic testing, and evaluation of noncomplement etiologies, particularly in the context of IC-MPGN. Addressing unmet needs in differential diagnosis and therapeutic options remains a priority, with ongoing research paving the way for potential targeted treatments that could revolutionize the management of these complex glomerular diseases.

## Disclosure

ASB has received consulting honoraria from Achillion, Alexion, Amgen, Apellis, Catalyst, Novartis, Q32 Bio, Silence, and Visterra. VC reports research grants from the NIH. FF has received consulting honoraria from Achillion, Alexion, Apellis, AstraZeneca, Novartis, Roche, and Sobi.

## Patient Consent

The cases discussed in this article are illustrative in nature and were derived from the authors' clinical expertise and experience. No patient data or personal health information are included in this article.
